# Treatment adherence among patients with hypertension: findings from a cross-sectional study

**DOI:** 10.1186/s40885-020-00151-1

**Published:** 2020-09-15

**Authors:** Fahad M. Algabbani, Aljoharah M. Algabbani

**Affiliations:** 1 Family Medicine Department, Prince Sultan Military Medical City (PSMMC), Riyadh, Riyadh Saudi Arabia; 2 George Mason University, Fairfax, VA USA

**Keywords:** High blood pressure, Uncontrolled hypertension, Nonadherence

## Abstract

**Background:**

Hypertension is a major risk factor for cardiovascular disease, which is the leading cause of mortality globally. Patient’s adherence to treatment is a cornerstone factor in controlling hypertension and its complications. This study assesses hypertension patients’ adherence to treatment and its associated factors.

**Methods:**

This cross-sectional study conducted in Riyadh, Saudi Arabia. The study targeted outpatients aged ≥18 years who were diagnosed with hypertension. Participants were recruited using a systemic sampling technique. The two main measurements were assessing adherence rate of antihypertensive medications using Morisky scale and identifying predictors of poor medication adherence among hypertensive patients including socio-economic and demographic data, health status, clinic visits, medication side effects, medications availability, and knowledge. Descriptive and logistic regression analyses were performed to assess factors associated with poor adherence.

**Results:**

A total of 306 hypertensive outpatients participated in this study. 42.2% of participants were adherent to antihypertensive medications. Almost half of participants (49%) who reported having no comorbidities were adherent to antihypertensive medications compared to participants with one or more than one comorbidities 41, 39% respectively. The presence of comorbid conditions and being on multiple medications were significantly associated with medication adherence (*P*-values, respectively, < 0.004, < 0.009). Patients with good knowledge about the disease and its complications were seven times more likely to have good adherence to medication (*P* <  0.001).

**Conclusions:**

Non-adherence to medications is prevalent among a proportion of hypertensive patients which urges continuous monitoring to medication adherence with special attention to at risks groups of patients. Patients with comorbidities and on multiple medications were at high risk of medication non-adherence. Patients’ knowledge on the disease was one of the main associated factors with non-adherence.

## Background

Hypertension is one of the most common chronic diseases in Saudi Arabia and a rising health burden, affecting 26.1% adult population [1]. Hypertension is a major risk factor for heart failure, myocardial infarction, cerebrovascular disease, and renal failure [2]. Controlling hypertension reduces the risk of cerebrovascular accident (CVA), coronary heart disease, congestive heart failure, and mortality [2, 3]. There are several factors that affect blood pressure control. Patients’ adherence to treatment is one of the major factors in controlling blood pressure and preventing hypertension complications [3]. The World Health Organization (WHO) defines adherence to long-term therapy as “the extent to which a person’s behavior-taking medication, following a diet, and/or executing lifestyle changes-corresponds with agreed recommendations from a healthcare provider” [3]. Patients with a high level of medication adherence were found to have better blood pressure control [4]. Still, adherence to hypertension treatment is challenging, due to the asymptomatic nature of the disease [5].

Several studies investigated the adherence rate among hypertension patients and sociodemographic factors affecting medication adherence including age, gender, comorbidities, patients’ knowledge about the disease, the number of medications. A study conducted in Saudi Arabia showed that only 34.7% of male hypertensive patients were found to be adherent to their medication [6]. The study reported a negative association between the presence of comorbidities and the adherence level [6]. A cross-sectional study on medication adherence among patients with hypertension in Malaysia, found an association between adherence and good knowledge of the medications and disease [7]. The study also found that the increase in the number of drugs patients taking has a negative effect on medication adherence [7]. Other studies had similar findings regarding the association between the number of medications and adherence [8–10]. In a cross-sectional study conducted in Iran, older patients reported high adherence to antihypertensive medication and better knowledge of their disease than younger patients [9]. However, number of studies reported no significant associations between age and medication adherence [8, 11]. Female patients were more likely to adhere to their medication, compared to males [12]. Another study on the prevalence and predictors of poor antihypertensive reported that male patients were more adherent than female patients [13]. Some studies reported no relationship between gender and adherence [9, 11].

Educational level and health literacy were shown to be associated with medication adherence. A cross-sectional study conducted in Iraq showed that adherence decreased in patients with primary and secondary school education, while no significant difference among patients with higher education and undereducated patients [14]. Similar results found in a systematic review conducted in hypertension management and medication adherence [15]. On the other hand, no association between educational level and adherence was found in a study conducted in Saudi Arabia [8]. However, good health knowledge of hypertension shown to be associated with good adherence to medication treatment in several studies [7, 11]. Two cross-sectional studies conducted in Turkey and Algeria showed a significant association between knowledge of complications related to hypertension and good adherence to antihypertensive therapy [16, 17].

Hypertension is one of the major health issues in Saudi Arabia; affecting more than a quarter of the Saudi adult population [1]. Only 37% of hypertensive patients on medication have their blood pressure controlled [18]. Non-adherence to antihypertensive medications is a potential contributing factor to uncontrolled hypertension. With limited studies conducted to investigate this challenging issue, this cross-sectional study aims to assess the adherence rate among hypertensive patients and associated factors affecting adherence to antihypertensive medications.

## Methods

### Study design and sampling

This cross-sectional study was conducted to determine the adherence rate of antihypertensive medications and the predictors of poor medication adherence among hypertensive patients at primary health clinics (PHCs) in Prince Sultan Medical City (PSMMC) in Riyadh the capital city of Saudi Arabia. Single population proportion formula was used to calculate the sample size based on the prevalence of hypertension in Saudi Arabia (26.1%) [1]. With a 95% confidence level and a 5% margin of error, a total of 306 randomly selected outpatients with hypertension following up at primary health clinics were included in this study.

### Participants recruitment

Participants in this study were recruited using a systemic sampling technique. Every fourth consecutive patient who fits the criteria was included. Arabic speaker patients older than 18 years who have been diagnosed with hypertension for more than three months were included in this study. Patients who do not speak Arabic had mental retardation, secondary hypertension, or who were younger than 18 years old were excluded from the study. The data was collected using a self-administered questionnaire that was distributed in the waiting area of the pharmacy. Illiterate participants were interviewed by a trained data collector. Before participants’ recruitment in the study, informed consent was obtained from all patients after a full explanation of the study. The study was approved and supervised by the institution review board (IRB) of Prince Sultan Medical City.

### Measurements

The questionnaire consists of four main sections. The first section assesses participants’ socio-economic factors including age, gender, marital status, occupation, the highest level of education currently attained, occupation, and monthly income. The second section assesses the factors affecting medication adherence including comorbidities, number of medications, number of daily doses, number of clinic visits, the distance to the clinic, medication side effects, medication availability at the pharmacy. The third section aimed to assess patients’ adherence to treatment. The fourth part assesses the patients’ knowledge about hypertension.

Medication adherence was assessed using the 8- items Morisky scale [19]. Morisky scale has been validated and found to be reliable (α = .83) [20]. The scale is based on the patients’ self-response and consists of eight questions, seven items with yes or no response, and one item with a 5-point Likert scale response option. The total score ranges from 1 to 8, the patient whose adherence score was six or more is considered adherent. For linguistic validation, questions were translated forward and backward into Arabic by an independent translator.

The participant’s knowledge about hypertension was assessed using nine structured questions. The questions focused on different aspects of hypertension, namely blood pressure target, lifestyle modification effect, complications, treatment, and cure. During analysis, patients who answered < 70% were considered to have low knowledge and patient how answered ≥70% of the questions were considered to have good knowledge. The 70 % cut-off is based on the minimally acceptable level of quality control at PSMMC [21].

The questionnaire was pre-tested and then a pilot survey was conducted on 20 patients for clarity and feasibility. The questionnaire was also evaluated and reviewed by two independent family medicine consultants at Prince Sultan Medical City for validation.

### Data analysis

Frequencies and percentages were used to assess participants’ characteristics. Chi-square analysis was used to determine the association between demographic, socioeconomic, and clinical factors with medication adherence. Logistic regression analysis was performed to assess factors associated with poor adherence. The variables were analyzed collectively using logistic regression to study the potential factors to avoid confounding bias. The association was considered statistically significant if the *P*-value was less than 0.05. Statistical Package for Social Sciences version 25 and Statistical Analysis System version 9.4 was used for data analysis.

## Results

A total of 306 outpatients who have hypertension participated in this study. Approximately 43% of the participants’ ages range was between 56 and 65. The majority of participants were married (92%), employed (61%), and had a high school diploma or above (80%). Most of the participants were middle income in the 5000–10,000 Saudi Riyal range of monthly income. Nearly one-third (28.1%) of the respondents in this study had no comorbidities while two-thirds reported having one or more comorbidities. The demographic, and socioeconomic clinical characteristics of the participants are presented in Table 1.

**Table 1:** Demographic, socioeconomic and clinical characteristics of patients

Variables	Categories	Frequency	Percentage
Age	Less than 45	39	12.7
	45–55	96	31.4
	56–65	132	43.1
	More than 65	39	12.7
Gender	Male	227	74.2
	Female	79	25.8
Marital status	Married	282	92.2
	Single	5	1.6
	Divorced	11	3.6
	Widow/ Widower	8	2.6
Educational level	College	76	24.8
	High school	170	55.6
	Middle school	14	4.6
	Elementary school	40	13.1
	Illiterate	6	2
Occupation	Public	89	29.1
	Military	90	29.4
	Private	8	2.6
	Retired	75	24.5
	Unemployed	44	14.4
Monthly income in Saudi Riyal	Less than 5000	82	26.8
	5000–9999	149	48.7
	10,000–14,999	47	15.4
	15,000–20,000	21	6.9
	More than 20,000	7	2.3
Comorbidity	No	86	28.1
	One	109	35.6
	More than one	111	36.3
Number of clinic visits in the last year	One	43	14.1
	Two	90	29.4
	Three	74	24.2
	Four	66	21.6
	More than four	33	10.8
Distance (driving time) from home to clinic	Less than 30 min	40	13.1
	30–60 min	209	68.3
	More than 60 min	57	18.6
Number of medication	Less than 4	210	68.6
	4 or more	96	31.4
Presence of side effects	Yes	60	19.6
	No	246	80.4
Stop taking medication when he/she is sick	Yes	15	4.9
	No	291	95.1
Stop taking medications when it is not available at the pharmacy	Yes	27	8.8
	No	279	91.2
Knowledge about hypertension	Good	155	50.7
	Poor	151	49.3

Only 13% of the respondents live at a distance of less than half an hour from the clinic. Of the total participants, 14.1% reported visiting the primary health clinic once in the last year, 29.4% twice, 24.2% three times. The majority of participants reported taking less than four medications a day and 31.4% reported taking four or more medications a day. As to antihypertensive medication side effects, 19.6% reported having medication side effects. Only 4.9% of the participants reported that they stop taking their antihypertensive medications when they get sick. Approximately 9% of the participants reported that they stop taking their medications when it is not available at the pharmacy. Findings show that half (50.7%) of the participants were knowledgeable about the disease. The clinical characteristics of the participants are presented in Table 1.

**Fig. 1:**
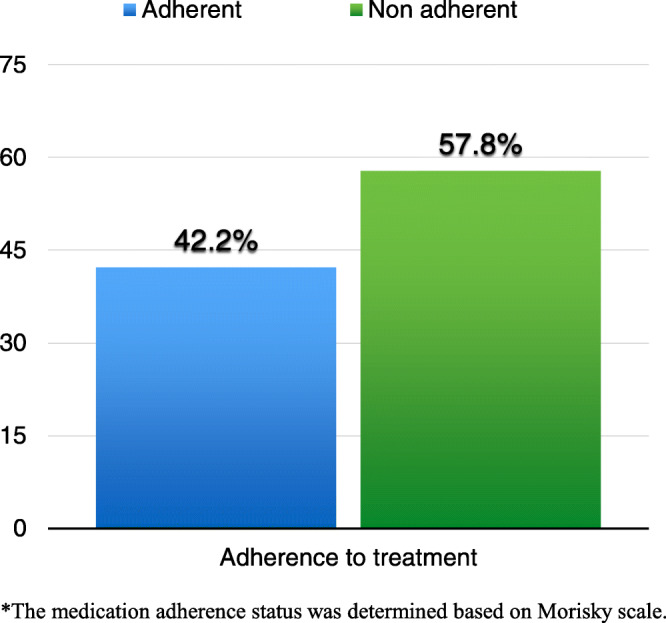
Distribution of patients according to their medication adherence status*

Figure 1 presents the percentage of participants’ adherence to antihypertensive treatment. Based on Morisky scale test results, 42.2% of the participants in this study were adherent to antihypertensive medications, while 57.8% were not adherent.

Table 2 presents the adherence rate in relation to the participants’ demographic, socioeconomic, and clinical characteristics. The presence of comorbid conditions is significantly associated with medication adherence (*P* <  0.001). Almost half of participants (49%) who reported having no comorbidities were adherent to antihypertensive medications compared to the participants with one or more than one comorbidities 41, 39% respectively. As for the number of medications, the adherence rate was found to be better among patients who were taking less than four medications (47.1%) compared to patients who were taking four or more medications (31.3%). Patients who visited the clinic once in the last year were more adherent than those who visited the clinic more than once (*p* < 0.05). No significant association between age, gender, income, educational level, and distance from home to the clinic.

**Table 2:** Sociodemographic, economic, and clinical characteristics of patients according to their medication adherence status

Variables	Categories	N Adherent (%)	N non-adherent (%)	*P* - value
Age	< 45	15 (38.5%)	24 (61.5%)	0.706
	46–55	45 (46.9%)	51 (53.1%)	
	54–65	54 (40.9%)	78 (59.1%)	
	> 65	15 (38.5%)	24 (61.5%)	
Gender	Male	99 (43.6%)	128 (56.4%)	0.382
	Female	30 (38%)	49 (62%)	
Marital status	Married	121 (42.9%)	161 (57.1%)	0.766
	Single	2 (40%)	3 (60%)	
	Divorced	3 (27.3%)	8 (72.7%)	
	Widow/Widower	3 (37.5%)	5 (62.5%)	
Educational level	Collage	30 (39.5%)	46 (60,5%)	0.937
	High school	73 (42.9	97 (57.1%)	
	Middle school	7 (50%)	7 (50%)	
	Elementary school	17 (42.5%)	23 (57.5%)	
	Illiterate	2 (33.3%)	4 (66%)	
Occupation	Public	34 (38.2%)	55 (61.8%)	0.487
	Military	44 (48.9%)	46 (51.1%)	
	Private	2 (25%)	6 (75%)	
	Retired	32 (42.7%)	43 (57.3%)	
	Unemployed	17 (38.6%)	27 (61.4%)	
Monthly income in Saudi Riyal	< 5000	38 (46.3%)	44 (53.7%)	0.599
	5000–9999	56 (37.6%)	93 (62.4%)	
	10,000–14,999	23 (48.9%)	24 (51.1%)	
	15,000–20,000	9 (42.9%)	12 (57.1%)	
	> 20,000	3 (42.9%)	4 (57.1%)	
Comorbidities	No comorbidities	49 (57%)	47 (43%)	0.004
	One	41 (37.6%)	68 (62%)	
	> 1	39 (35.1%)	72 (64.9%)	
Number of medication	< 4	99 (47.1%)	111 (52.9%)	0.009
	≥ 4	30 (31.3%)	66 (68.8%)	
Presence of side effects	Yes	19 (31.7%)	41 (68.3%)	0.066
	No	110 (44.7%)	136 (55.3%)	
Stop taking medications when he/she’s sick	Yes	3 (20%)	12 (80%)	0.75
	No	126 (43.3%)	165 (56.7%)	
Stop taking medications when it is not available at the pharmacy	Yes	7 (25.9%)	20 (74.1%)	0.074
	No	122 (43.7%)	157 (56.3%)	
Distance from home to the clinic	< 30 min	16 (40%)	24 (60%)	0.276
	30–60 min	94 (45%)	115 (55%)	
	> 60 min	19 (33.3%)	38 (66.7%)	
Number of clinic visits in last year	One	25 (58.1%)	18 (41.9%)	0.044
	Two	42 (46.7%	48 (53.3%)	
	Three	26 (35.1%)	48 (64.9%)	
	Four	27 (40.9%)	39 (59.1%)	
	< 4	9 (27.3%)	24 (72.7%)	
Knowledge about hypertension	Good	89 (57.4%)	66 (42.6%)	< 0.005
	Poor	40 (26.5%)	111 (73.5%)	

The participants were asked eight questions about hypertension. Table 3 shows the distribution of the correct and incorrect answers giving by the participants. Most participants (64.4%) knew the target blood pressure for hypertensive patients and 40.8% think that hypertension can be cured. The majority (78%) knew that a low salt diet helps in lowering high blood pressure. Only 61% knew that hypertension can affect eyes and 13.4% reported that they stop taking their medication when they feel their blood pressure under control.

**Table 3:** The patients’ responses to hypertension knowledge questions

Assessment items	N correct (%)	N Incorrect (%)
The target blood pressure level for hypertensive patients	188 (64.4%)	118 (38.6%)
Can hypertension be cured?	181 (59.2%)	125 (40.8%)
Exercise help in controlling blood pressure	285 (93.1%)	21 (6.9%)
Reducing salt intake help in controlling blood pressure	241 (78.8%)	65 (21.2%)
High blood pressure may affect the heart?	289 (94.4%)	17 (5.6%)
High blood pressure may affect the brain?	273 (77.5%)	69 (22.5%)
High blood pressure may affect the kidneys?	265 (86.6%)	41 (13.4%)
High blood pressure may affect the eyes?	188 (61.4%)	118 (38.6%)
Stop taking medications when you feel like your blood pressure or your symptoms are under control?	264 (86.3%)	42 (13.7%)

**Fig. 2:**
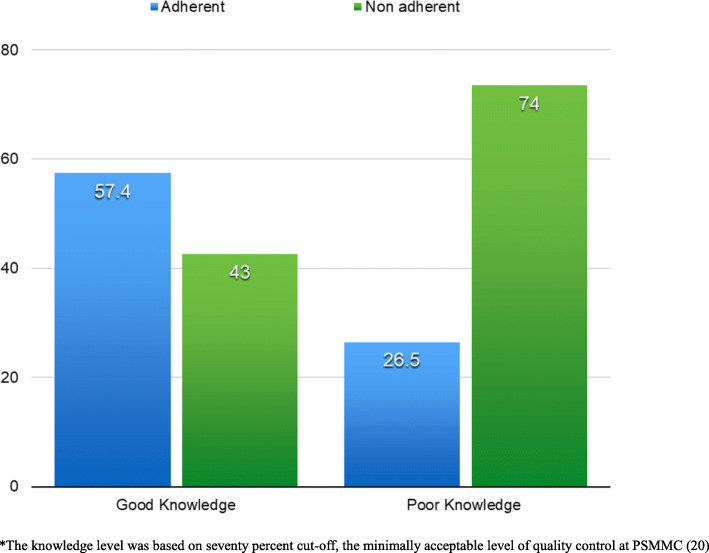
Adherence to hypertension treatment among patients with good and poor knowledge level*

Adherence to antihypertensive medications among patients with good and poor knowledge levels about hypertension was assessed based on nine structured questions. Figure 2 shows the adherence to hypertension treatment among patients with good and poor knowledge level. 57.4% of patients with good knowledge levels were adherent compared to 42.6% who were not adherent. The majority (73.5%) of patients with poor knowledge levels were not adherent to treatment.

Table 4 presents the factors associated with good adherence. When conducting binary logistic regression, knowledge about the disease was found to be significantly associated with adherence. Patients with good knowledge about the disease were seven times more likely to have good adherence to antihypertensive medications than those with poor knowledge (AOR 7.4 [95% CI: 4.177–13.121], *p* < 0.001).

**Table 4:** Logistic regression results of factors associated with good medication adherence

Variables	AOR	P-value	95% Confidence interval	
Age	1.204	0.255	0.875	1.657
Gender	1.314	0.401	0.695	2.483
Education	0.766	0.096	0.559	1.049
Knowledge about hypertension	7.403	< 0.001	4.177	13.121
Daily dosing	1.214	0.190	0.908	1.621

## Discussion

Several studies have investigated factors affecting medication adherence. This study shows that the level of adherence to antihypertensive medications is low. In this sample the adherence rate to hypertension treatment was found to be only 42%, which is similar to the study conducted in Al-Khobar and higher than the study conducted in Taif where adherence rate was found to be 47 and 34.7%, respectively [6, 8]. Other studies conducted in different countries reported adherence rates ranging from 15 to 88% [22–25]. This discrepancy in adherence rate is potentially due to the differences in population characteristics, medication adherence assessment tools, and healthcare systems.

The association between sociodemographic and socioeconomic factors and adherence level has been investigated in several studies. In a study done in Hong Kong, older patients were found to be more adherent. However, in this study, there was no association between age and adherence. In another study done in the United States, female patients were less adherent to hypertension medication compared to male patients [13]. A study conducted in Malaysia reported that female patients were more adherent than male patients [22]. Our study showed that there was no significant relationship between gender and adherence. A meta-analysis suggested that the association between age, gender, and adherence level is weak [26]. The results of our study also demonstrate no significant relationship between marital status and educational level with adherence, which is similar to findings reported by other studies [9, 27].

Previous research found that shorter traveling time from residence to the healthcare facility could increase patients’ adherence [28]. A study in Ethiopia found that the adherence level was lower in patients who lived more than 10 km from healthcare facilities [29]. A cross-sectional observational study done in Northwest Ethiopia indicated that patients who live less than 10 km from the healthcare facility had an adherence rate of 74% compared to 58% for patients who live far from the healthcare facility [29]. As the authors attributed this problem to poor infrastructure and lack of transportation in Ethiopia, the study suggested that shorter traveling time from residence to the healthcare facility could increase patients’ adherence [29]. In this study, distance from home to the clinic was not associated with hypertensive treatment. These differences may be due to the higher level of car ownership in Saudis Arabia which makes it easier to access health care facilities [30].

Only 8.8% of the participants reported not taking their medication when it is not available at the hospital pharmacy. This low percentage may be explained by the multiple community health centers in Saudi Arabia which provide free health care including medications dispensing. Moreover, the medication cost at private pharmacies in Saudi Arabia is affordable for most patients. According to the published Saudi Hypertension Management Guidelines the prices of the antihypertensive medications ranges between 7 to 118 Saudi Riyal (about 2 to 31 US Dollar) [31].

Many patients with hypertension will need two or more antihypertensive medications to achieve goal blood pressure [2]. In this sample significant association was observed between the number of medications and adherence level. The adherence rate among patients taking less than four medications was 47.1% compared to 31.3% to those who take four or more medications. Similarly, other studies reported the negative association between the number of medication and adherence levels [29, 32].

Findings indicate that patients with multiple comorbidities were less adherent to antihypertensive medication, which is inconsistent with a previous study done in Taif which showed a negative association between the presence of comorbidity and adherence level [6]. This may be related to the fact that most patients with multiple comorbidities require taking multiple complex medications.

The result of our study showed that the patient who visited the clinic once in the last year were more adherent than the patient who visited the clinic more than once. This could be explained by that most patients with multiple comorbidities and on multiple medications frequently visit the clinic for issues related to their disease and to refill prescriptions.

Our study demonstrated the positive association between knowledge and adherence levels. Patients who had good knowledge were more adherent to the treatment [29]. Previous studies showed that patients who know the ideal target blood pressure level were more adherent to their medications [16, 20]. In this study, only 64.4% of the participants knew the ideal target of blood pressure and 40.8% of the patients believe that hypertension can be cured. A study conducted in Rajshahi, Bangladesh found that 65.8% of the patients believe that hypertension is curable. Patients how have been educated by their physicians and healthcare providers were more adherents to treatment as they have a better understanding of the disease nature, the ideal target of blood pressure, and the complications of hypertension [33]. Therefore, patient education in disease nature and management is considered a key factor in the treatment of hypertension.

The study findings were based on self-reported survey. Self-reported data is a common method used in a cross-sectional study. However, self-reported data is subjected to biases such as response and recall biases that can lead to under- or overestimation of findings. On the other hand, adherence in this study was measured based on a validated self-report adherence scale and knowledge was tested based on evaluated and reviewed assessment items by two independent family medicine consultants. Moreover, this study conducted in one of the largest medical cities that serves a large community in the capital city Riyadh. Due to the study design and sampling method, study findings cannot be generalized and temporal relationships cannot be established between risk factors and adherence. Nevertheless, this study provides a snapshot of adherence to antihypertensive medication status and associated determinates among outpatients. Future large scale longitudinal studies will contribute to a better understanding of adherence status and associated factors among hypertensive patients.

## Conclusions

Non-adherence to medications is prevalent in proportion of patients with hypertension. Therefore, there is an urge to continually monitor patients’ adherence to antihypertensive medication using a standardized scale. Patients with comorbidities and on multiple medications were at higher risk of non-adherence. There is a need to encourage patients on multiple medications to use adherence aids such as weekly pill organizers and medication alarm devices. Hypertensive patients’ knowledge of the disease and its complications was one of the main factors affecting patients’ adherence to treatment. Implementation of health awareness interventions and education programs intended for hypertensive patients will help improve medication adherence.

## Data Availability

The data sets generated during and/or analyzed during the current study are available from the corresponding authors on reasonable request.

## Abbreviations

AOR: Adjusted odd ratio
OR: Odd ratio
CI: Confidence interval
BP: Blood pressure
P: *P* value
PSMMC: Prince Sultan Medical City
PHC: primary health clinic
IRB: institution review board
CVA: Cerebrovascular accident
WHO: The World Health Organization
